# Classification of substandard factors in perinatal care: development and multidisciplinary inter-rater agreement of the Groningen-system

**DOI:** 10.1186/s12884-015-0638-5

**Published:** 2015-09-11

**Authors:** Mariet Th. van Diem, Albertus Timmer, Sanne J. Gordijn, Klasien A. Bergman, Fleurisca J. Korteweg, Joke Ravise, Ellen Vreugdenhil, Jan Jaap H.M. Erwich

**Affiliations:** Department of Obstetrics and Gynaecology, University Medical Centre, University of Groningen, PO BOX 30.001, CB20 9700 RB Groningen, The Netherlands; Department of Pathology, University Medical Centre, University of Groningen, Groningen, The Netherlands; Department of Neonatology, University Medical Centre, University of Groningen, Groningen, The Netherlands; Department of Obstetrics and Gynaecology, Martini Hospital, Groningen, The Netherlands; Independent Midwives Practice Hoogezand, Hoogezand, The Netherlands

## Abstract

**Background:**

Perinatal audit is an established method for improving the quality of perinatal care. In audit meetings substandard factors (SSF) are identified in cases of perinatal mortality and morbidity. To our knowledge there is no classification system specifically designed for the classification of substandard factors. Such a classification may help to standardise allocation of substandard factors to categories. This will help to prioritise, guide and implement actions in quality improvement programs.

**Methods:**

A classification system of 284 substandard factors (SSF) identified in perinatal audit meetings between 2007 and 2011 was drawn up using the WHO Conceptual Framework for the International Classification for Patient Safety as a starting point. Discussions were held on inter-rater disagreements, inclusion of items, format and organisation and definitions of the main- and subcategories. A guideline was developed. An independent multidisciplinary group tested the classification. Independent of inter-rater agreement the allocations to categories were counted. For the counts in the subcategories one and two, we used the allocations in the main category as reference. The chance corrected agreement between classifiers was tested with Cohen's kappa statistic.

**Results:**

The classification consists of 9 main categories with one or two subcategories. The main categories are (1) Equipment and Materials, (2) Medication, (3) Additional tests/ investigations, (4) Transportation , (5) Documentation, (6) Communication, (7) Medical practice, (8) Other and (9) non classifiable. Of 3663 allocations by 13 classifiers 1452 SSF’s were allocated (40 %) to ‘medical practice’ and 1247 (34 %) to ‘documentation’. 118 (3 %) times SSF’s were not classifiable, mainly due to unclear phrasing of the SSF. The chance corrected agreement of 284 substandard factors in the main category was 0.68 (95 % CI 0.66-0.70) and 0.57 (95 % CI 0.54-0.59) for the CDG and the IGD respectively.

**Conclusions:**

Classifying substandard factors has given insight into problem area's in perinatal care and can give direction to medical, political and financial quality improvement measures.

The Groningen-system has well defined categories and subcategories and the guidelines and examples are clear. The multidisciplinary inter-rater agreement is moderate to good. Improvement of the phrasing of the substandard factors is expected to improve inter-rater agreement.

**Electronic supplementary material:**

The online version of this article (doi:10.1186/s12884-015-0638-5) contains supplementary material, which is available to authorized users.

## Background

Perinatal audit is an established method for improving the quality of perinatal care in the Netherlands since 2010. In an audit meeting substandard factors (SSF) are defined in cases of perinatal mortality and morbidity [1]. To our knowledge there is no classification system specifically designed for the classification of substandard factors. Such a classification may help to standardise allocation of substandard factors to categories, prioritise, guide and implement actions in quality improvement programs.

In patient safety and quality of healthcare systems several terms are used to describe unwanted events in the care process. An often used term is “adverse event”, which is defined as an unintended injury that results in temporary or permanent disability, death or prolonged hospital stay. An adverse event is caused by healthcare factors rather than by the patient's underlying disease process [2]. Other authors use the term “incident”, which is defined as an unintended event during the care process that resulted, could have resulted or still might result in harm to the patient [3]. A substandard factor, however, is defined as a care management problem that involves care that deviates from the safe limits of practice as laid down in guidelines, standards, protocols or normal practice and has the potential to lead, directly or indirectly, to an adverse outcome for the patient [1, 4]. This concept differs intrinsically from the concept of adverse events and incidents, because it specifically mentions the standards that are used to assess the care process.

In perinatal audit meetings in obstetric cooperation units, SSF are analysed using a model based on the root cause analysis that was first introduced by Reason [5]. As a result of the analysis, actions are formulated to prevent the SSF from reoccurring. This may lead to the improvement of care at a local level.

At a national level insight into the relationship between SSF’s in perinatal care and characteristics of cases or the care process in which they occurred, is needed. With this insight the efficiency of quality management can be improved in several ways. Medically, by giving direction to the development of medical guidelines by the professional organisations, politically by giving direction to the organisation of perinatal care and financially by giving direction to funding of quality improvement activities and research.

A classification system aids uniformity and comparison of results at a national and international level and should therefore have a structure that allows unambiguous allocation to representative categories of substandard factors to ensure a high percentage of classified and a low percentage of unclassified SSF’s [6]. Clear definitions of categories as well as guidelines are necessary to facilitate the allocation to the categories and easy use by a multidisciplinary team.

Our aim was to develop a classification system for SSF’s, that describes the categories of substandard factors and that will facilitate the analysis of the relation between SSF’s and different characteristics of perinatal mortality and morbidity such as cause of death, socio-economic status or maternal disease. In this paper we describe the development of the Groningen-system for the classification of substandard factors. We also assess the inter-rater agreement in a multidisciplinary setting.

## Methods

### Classification development group (CDG)

The classification development group consisted of a perinatologist, a paediatrician, two registrars in obstetrics and gynaecology, a midwife researcher/epidemiologist, a primary care midwife, a perinatal pathologist, and a data manager, with various levels of experience in the development of classifications. Five (FJK, SJG, JJE, JMR, KAB) of them have developed a classification system before and are experienced classifiers [6].

### Independent classification group (ICG)

The independent classification group consisted of an obstetrician, a paediatrician, a secondary care midwife, an independent primary care midwife and an obstetric nurse. As caregivers the members of the ICG were familiar with perinatal audit and the concept of substandard factors, but they had limited experience with classifications.

### Data

157 substandard factors, identified during 64 unit based perinatal mortality audit meetings in 15 perinatal cooperation units from September 2007 till March 2010 (IMPACT project) and 127 substandard factors identified during monthly unit based audit meetings at the University Medical Centre Groningen from March 2010 till September 2011, were used for the development of the classification [1]. In these audit meetings the care providers of an obstetric cooperation unit (gynaecologists, midwives, paediatricians, pathologists, general practitioners, obstetric and paediatric nurses, geneticists) discussed anonymous narratives of cases of perinatal mortality. The care process and the circumstances under which the mortality occurred were outlined in a set narrative format. Identified SSF’s were formulated by the group and written down as free text by one of the members of the audit group at a time that there was no classification system available. The purpose of these meetings was and is to improve care and not to blame individual care providers. Case notes were not available, since audit meetings are confidential and substandard factors are filed separately from the case notes.

### Development process

The starting point for the classification was the database with 284 SSF’s and “The Conceptual Framework for the International Classification for Patient Safety” developed by the World Health Organisation and reported on by Runciman [7]. In addition the local format for the Safe Reporting of (near) Incidents at the University Medical Centre Groningen was used.

In nine sessions the classification format was developed. For each session the CDG classified a random sample of the 284 substandard factors according to each new version of the classification. In the sessions multidisciplinary discussions were held on inter-rater disagreements and ambiguous allocations, items that should or should not be included in the classification, the format and organization of the classification and the definitions of the main categories and the subcategories. A major discussion was held on the subject of hierarchy. Most SSF’s are part of the care process and could just as easily be classified under the specific item (e.g. medication or tests/investigation) as under medical practice (e.g. management plan/management). This would violate the unambiguous allocation to representative categories of SSF and consequently violate our goal to make a classification that facilitates the analysis of the relation between SSF’s and characteristics of the cases and their care process. We therefore decided that a strict hierarchy would be preferable. To accommodate this hierarchy the classification is ordered from very specific to broad aspects of the care process as they occurred in the list of 284 SSF.

Table 1 shows the 9 main categories with subcategories (Table 1). The definitions of the terms used and the allocation rules were drawn up in a guideline. Examples of SSF’s and their classification are shown Additional file 1.

**Table 1:** The number of allocations of substandard factors in categories and subcategories by 13 classifiers^a^

	Main category	n^b^	%	Subcategory 1	n^c^	%	Subcategory 2	n^d^	%
	Allocations	3663		Allocations	26				
1	Equipment and Materials	29	(1)	1. use	2	(7)			
				2. performance	5	(17)			
				3. availability	15	(52)			
				4. other	4	(14)			
				Allocations	13				
2	Medication	14	(<1)	1. substance itself	-	-			
				2. dosage	10	(71)			
				3. administration	-	-			
				4. other	3	(21)			
				Allocations	297				
3	Additional tests/ investigations	326	(9)	1. requestform	58	(18)			
				2. labelling patient material	23	(7)			
				3. dispatch patientmaterial	9	(3)			
				4. execution	85	(26)			
				5. interpretation testresult by test performer	29	(9)			
				6. testresult to person who requested	47	(14)			
				7. other	46	(14)			
				Allocations	35				
4	Transportation	39	(1)	1. home-hospital	15	(38)			
				2. between hospitals	1	(3)			
				3. within the hospital/department/ward	9	(23)			
				4. other	10	(26)			
				Allocations	1121				
5	Documentation	1247	(34)	1. basic data	406	(33)			
				2. observations/examinations	270	(22)			
				3. considerations/management	314	(25)			
				4. other	131	(11)			
				Allocations	311				
6	Communication	348	(10)	1. same echelon, equal level	44	(13)			
				2. same echelon, different level	24	(7)			
				3. different echelons	104	(30)			
				4. with patient	71	(20)			
				5. between departments	18	(5)			
				6. other	50	(14)			
				Allocations	1449		Allocations	450	
7	Medical practice	1452	(40)	1. diagnosis	467	(32)	*1. use of guidelines*	311	(67)
							*2. content of guidelines*	3	(1)
							*3. common practice*	136	(29)
							Allocations	878	
				2. management plan/management	911	(63)	*1. use of guidelines*	588	(65)
							*2. content of guidelines*	32	(4)
							*3. common practice*	258	(28)
				3. other	71	(5)			
8	Other	90	(2)						
9	Non classifiable	118	(3)						
	Total	3663		Total number of allocations	3252		Total number of allocations	1328	

^a^8 classifiers in the Classification Development Group, 5 classifiers in the Independent Classification Group

^b^n is the total number of allocations within the main category. On 29 occasions SSF were not allocated to a main category, resulting in a the total number of allocations of 3663 instead of 284*13 = 3692

^c^n is the total number of allocations within subcategory 1, grouped by the allocations in the main category

^d^n is the total number of allocations within subcategory 2, grouped by the allocations in the main category and subcategory 1

In the final stages of the development process all 284 SSF were classified by the CDG. Furthermore the independent classification group (ICG) classified all 284 SSF after a short training and using the guidelines, agreements and examples.

### Guideline to the classification

#### Equipment and Materials

Allocation to this category is justified if the equipment and materials for monitoring, diagnosis or treatment are not or cannot be used as intended. The equipment, being the basic machinery or tools, or the materials -usually disposable in nature and for single use- for monitoring, diagnosis or treatment. The subcategories include (1.1) use: the equipment and materials are available, but are not used; (1.2) performance: the equipment and materials are available, but are not functioning as intended; (1.3) availability: the equipment and materials are not ready for use or not available; and (1.4) other: the SSF involves materials and equipment, but cannot be classified in 1.1 to 1.3.

#### Medication

Allocation to this category is justified if the guidelines for the administration of medication in a given clinical situation exist but were not followed. In this category the medication itself and not its indication for prescribing is classified. The subcategories include (2.1) substance itself: incorrect medication was administered; (2.2) dosage: the recommended dosage was not administered; (2.3) administration: the recommended route for administration was not used; and (2.4) other: the SSF involved medication, but cannot be classified in 2.1 to 2.3.

#### Tests/investigations

Allocation to this category is justified if tests and/or investigations are not executed and processed according to guidelines or common practice. In this category the test or investigation itself and not its indication for performing it, is classified. The subcategories include (3.1) the test/investigation request: recommendations for requests for tests and/or investigations are not followed; (3.2) labelling patient material: recommendations for labelling of patient material for tests and/or investigations are not followed; (3.3) transport patient material: recommendations for transport of patient material for tests and/or investigations are not followed; (3.4) execution of tests/investigations: The execution of tests and/or investigations is not performed according to guidelines or common practice; (3.5) interpretation of the result by the performer of the test/investigation: the results of tests/investigations are incorrectly interpreted by the person performing the test or investigation; (3.6) Results of tests/investigations to the person who requested them: the results of tests/investigations are not processed according to guidelines or common practice; (3.7) other: the SSF involves a test or investigation, but cannot be classified in 3.1 to 3.6.

#### Transportation

Allocation to this category is justified if the transportation of a patient is not performed according to the recommendations, local protocols or common practice. The subcategories include (4.1) from home to hospital: the recommendations for transportation of a patient to the hospital in time and/or mode are not followed; (4.2) between hospitals: the recommendations for transportation of a patient between hospitals in time and/or mode are not followed; (4.3) within the hospital: the recommendations or common practice for transportation of a patient within the hospital in time and/or mode are not followed; (4.4) Other: the SSF involves transportation of the patient, but cannot be classified in 4.1 to 4.3.

#### Documentation

Allocation to this category is justified if the documentation in the patient record is not sufficient to facilitate the best possible patient care. The subcategories include (5.1) basic data: documentation in the patient record of basic data is not sufficient or absent. Basic data are routinely collected data (general particulars, family-, medical- and obstetric history and the present pregnancy) at the first and subsequent antenatal visits, the natal and postnatal period; (5.2) observations/examinations: documentation of the observations and or examination on the physical and psychosocial state is not sufficient or absent; (5.3) considerations/management: documentation in the patient record of the considerations leading to patient management and the choice of patient management in a given clinical situation is insufficient or absent; (5.4) other: the SSF involves documentation, but cannot be classified in 5.1 to 5.4.

#### Communication

Allocation to this category is justified if communication between care givers and/or the patient is not sufficient to facilitate the best possible patient care. The subcategories include (6.1) same echelon and equal level: the communication between professionals of an equal professional level within the same echelon was insufficient; (6.2) same echelon, different level: the communication between professionals of a different professional level within the same echelon was insufficient; (6.3) different echelon: the communication between professionals in different echelons was insufficient; (6.4) with the patient: the communication with the patient was insufficient; (6.5) between departments: the communication between departments within a healthcare facility was insufficient; (6.6) other: the SSF involves communication, but cannot be classified in 6.1 to 6.5.

#### Medical practice

Allocation to this category is justified if the best possible medical patient care is not given in the different stages of the care process. The subcategories include (7.1) diagnosis: the recognition and diagnosis of the presence or absence of a disease or a complication takes longer then can be expected, with subcategories in (7.1.1) use or (7.1.2) content of guidelines and (7.1.3) common practice; (7.2) making/execution management plan: the management plan is not made or executed after the diagnosis of the presence or absence of a disease or a complication or takes longer than can be expected, with subcategories in (7.2.1) use or (7.2.2) content of guidelines and (7.2.3) common practice; (7.4) other: the SSF involves medical practice, but cannot be classified in 7.1 to 7.3.

#### Other

The SSF is clear, but cannot be classified in any of the earlier categories.

#### Not classifiable

The SSF is phrased in such a way that classification is impossible.

### Agreements on classification

Certain SSF or their presentation led to discussions and not to consensus, therefore additional agreements were prepared for use of the classification:

1. A SSF can never be caused by a patient, although the patient sometimes appears to be the main causal factor in the occurrence of the substandard factor. Inherent to its definition a SSF is a care management problem and therefore always related to the care process provided by the caregivers working in a particular health care system. Although this concept is clear, the patient factor seemed too important to ignore altogether in the classification. Therefore it was agreed upon to classify this factor under (8) Other.
2. If a patient had her booking for maternity care later in pregnancy than normal, the SSF was “late for booking for maternity care”. This is considered to be a medical practice problem, because a management plan for the early weeks of pregnancy was not made or executed. Although knowing the cause of a SSF (i.e. the patient did not come for intake) is important for actions to prevent this from happening again, the cause of this SSF is of no consequence for its classification.
3. If a certain action is not documented it is usually assumed that the action is not performed. However, if it is reasonable to assume that the action was performed but not documented, this substandard factor is classified as “documentation” rather than not performed. For example: The standard neonatal examination is not documented. If half an hour after birth the baby is referred to the paediatrician for anal atresia it can be assumed that the neonatal examination was performed but not documented.

### Analysis

Independent of inter-rater agreement the allocations to categories were counted. For the counts in the subcategories one and two, we used the allocations in the main category as reference, again independent of inter-rater agreement.

For the description of the uncorrected agreement it was agreed upon by the CDG that one person of the group disagreeing in a classifier group in the allocation of a SSF to a category was acceptable (CDG:7/8 being 88 % and ICG:4/5 being 80 % agreement). For the two groups together (13 classifiers) it was agreed upon that two disagreements in the allocation of a SSF to a category was acceptable (85 % agreement). For the uncorrected and chance corrected agreement(κ) for allocations to the subcategories one and two, agreement in the preceding categories had to be 100 %.

### Statistical methods

For descriptive analyses we used Microsoft Excell 2010 and IBM SPSS statistics 19 and for the analyses of the multi-rater chance corrected agreement (Cohen’s kappa statistic) [8] IBM SPSS statistics 19 with added macro mkappasc [9]. For the interpretation of the kappa statistic we used the following categories: <0.20 poor, 0.21–0.40 fair, 0.41–0.60 moderate, 0.61–0.80 good and 0.81–1.00 very good agreement [10]. Confidence intervals (95 %) were calculated for Cohen’s kappa statistic.

## Results

Table 1 shows the number of times the two classification groups (CDG and ICG) allocated the SSF’s to the main categories and the associated subcategories irrespective of inter-rater agreement (Table 1). 13 classifiers (CDG (n = 8) and ICG (n = 5)) classified 284 SSF’s. 29 times SSF were not allocated to a main category resulting in a total number 3663 allocations. These were random omissions.

1452 SSF’s were allocated (40 %) to ‘medical practice’ and 1247 (34 %) to ‘documentation’. 118 (3 %) times SSF’s were not classifiable, mainly due to unclear phrasing of the SSF.

In subcategory one of ‘medical practice’(1449 allocations) SSF’s were allocated 911 (63 %) times to ‘making of a management plan and its execution’. In subcategory one of ‘documentation’ (1121 allocations) SSF’s were allocated 406 (33 %) times to ‘documentation of the basic data in the patient record’ and 314 (25 %) times to the ‘documentation in the patient record of the considerations leading to patient management and the choice of patient management’.

In subcategory two of the categories ‘medical practice’, ‘diagnosis, management plan, other‘ SSF’s were allocated 588 (65 %) times to the ‘use of guidelines’ in the category management plan/management.

The acceptance of one disagreement in the two classifier groups resulted in an 88 % and 80 % agreement score in the CDG (8 classifiers) and ICG (5 classifiers) respectively. For the two groups together this resulted in an 85 % agreement score.

The uncorrected agreement of 284 substandard factors on the allocations in the main category was 194 (68 %) and 189 (67 %) for the CDG and the ICG respectively and 177(62 %) for the two groups combined. Table 2 shows the uncorrected agreement for the subcategories one and two and Table 3 shows the uncorrected agreement divided over the main category (Tables 2 and 3). The disagreement was mainly due to unclear phrasing of the SSF’s and not following the guidelines.

**Table 2:** Uncorrected agreement on the classification of 284 SSF’s in main categories and subcategories of the classification of substandard factors, classified by two classifier groups (CDG* and ICG^¥^) separately and combined

	CDG (88 % agreement)^£^		ICG (80 % agreement)^£^		CDG and ICG (85 % agreement)^£^	
	n^§^	n(%)	n^§^	n(%)	n^§^	n(%)
Main category	284	194(68)	284	189(67)	284	177(62)
Subcategory 1	140	61(44)	111	54(49)	87	15(17)
Subcategory 2	14	11(79)	14	8(57)	6	0 (0)

^£^One disagreement on allocation per group acceptable, i.e. CDG 7 out of 8, ICG 4 out of 5. Two disagreements on allocation in CDG and ICG together acceptable, i.e. 11 out of 13

^*^CDG = Classification Development Group (8 allocators)

^¥^ICG = Independent Classification Group (5 allocators)

^§^n = total number of substandard factors (main categories). For subcategories : only the substandard factors with 100 % uncorrected agreement in the main category were used (CDG 140/194). For subcategory 2: only the substandard factors with 100 % uncorrected agreement in main category “medical practice” and in subcategory 1 were used (CDG 14/61). The same principle was used for ICG and ICG + ICG together

**Table 3:** Uncorrected agreement on the classification of 284 substandard factors classified by two classifier groups (CDG^a^ and ICG^b^) separately and combined, divided over the main categories of the classification of substandard factors

	CDG 86 % agreement^c^		ICG 80 % agreement^c^		CDG and ICG 85 % agreement^c^	
	n	(%)	n	(%)	n	(%)
Equipment and Materials	1	(1)	1	(1)	1	(1)
Medication					-	-
Additional tests/investigations	13	(7)	7	(4)	6	(3)
Transportation	3	(2)	2	(1)	2	(1)
Documentation	87	(45)	86	(46)	85	(48)
Communication	15	(8)	13	(7)	13	(7)
Medical Practice	75	(39)	79	(42)	70	(40)
Other	-				-	-
Non Classifiable	-		1	(1)	-	-
Total	194	(100)	189	(100)	177	(100)

^a^CDG = Classification Development Group

^b^ICG = Independent Classification Group

^c^One disagreement on allocation per group acceptable, i.e. CDG 7 out of 8, ICG 4 out of 5. Two disagreements on allocation in CDG and ICG together acceptable, i.e. 11 out of 13

The chance corrected agreement(κ) of 284 SSF’s on the allocations in the main category was 0.68 (95 % confidence interval 0.66-0.70) and 0.57 (95 % confidence interval 0.54-0.59) for the CDG and the IGD respectively. Table 4 shows the chance corrected agreement for the two groups combined and the subcategories one and two (Table 4).

**Table 4:** Chance corrected agreement on the classification of 284 substandard factors in the main category and subcategories of the Groningen classification of substandard factors, classified by two classifier groups (CDG* and ICG¥) separately and combined

	CDG		ICG		CDG and ICG	
	n^§^	κ (95 % CI)*	n^§^	κ (95 % CI)*	n^§^	κ (95 % CI)*
Main category	284	0.68 (0.66-0.70)	284	0,57 (0.54-0.59)	284	0.64 (0.62-0.65)
Subcategory1	140	0.56 (0.54-0.59)	111	0.44 (0.39-0.48)	87	0.50 (0.48-0.52)
Subcategory 2	45	0.28 (−0.09-0.66)	27	0.37 (−0.03-0.72)	15	−0.01 (−0,38-0,36)

*CDG = Classification Development Group

^¥^ICG = Independent Classification Group

^§^n = total number of substandard factors (main category) and number of substandard factors with 100 % agreement in the main category en subcategory1 respectively

*κ (95 % CI) = kappa statistic ( 95 % confidence interval)

## Discussion

Perinatal audit with a methodical analysis of the care process resulting in the identification of substandard factors was introduced in the Netherlands in 2010 [11] Although similar analytical systems exist in the medical field [12] the term and concept of substandard factor is new and differs from incident or adverse event [2]. In this paper we describe the development of the Groningen-system for the classification of substandard factors. This process was initiated by the substandard factors identified in perinatal audit meetings and the lack of an existing classification. For the main categories the chance corrected agreement was good in the CDG and moderate in the ICG. For the subcategories one and two this was moderate and fair respectively. In the allocation counts and the uncorrected agreements, documentation and medical practice were the most prominent area’s to which SSF were allocated.

As a result of audit meetings local perinatal cooperation units in the Netherlands can improve the quality of their care. Although there are initiatives at a regional and national level, the use of the aggregated audit results is complicated [13] and therefore the potential for the improvement of the quality of perinatal care and future research is not yet fully used.

Up to now, substandard factors identified in audit groups are not allocated to different clearly [14] defined categories according to guidelines [11]. Consequently, there is a risk of misclassification of SSF’s and inferences may be incorrect [14]. This may lead to inappropriate or unnecessary actions and allocation of funds. In addition, the potential of a database with continuously growing categories of substandard factors cannot be used as a data source for future research.

A large database of substandard factors allowed us to develop this first classification system for substandard factors. Classification systems are preferably short, simple and easy to use with a low percentage of unclassifiable cases [15]. It can be argued that the Groningen system is too complex with its nine main categories and three to seven subcategories. The representation of both detailed and general aspects of the care process in the classification, however, makes improvement of care possible at different levels. This complexity also facilitates the detailed investigation of the relation between SSF and characteristics of the cases or other aspects of the care process and helps to focus on specific categories of SSF. After investigation of the common underlying causes this may lead to a variety of initiatives to improve care, such as changes in the initial and the advanced training of caregivers, the implementation process of guidelines or the information technology used in medical care. In addition it facilitates comparison of the occurrence of SSF’s at a national and international level.

This classification system was developed in a high-income country. In low-income countries the distribution of SSF may be different where, for example, transportation resulting in delay to obtain appropriate care is more common. We are convinced that, by adding specific factors, our system can easily be adjusted to these circumstances while keeping the basic principles of the classification –hierarchy, detailed and general aspects of care- intact. In addition, the categories of our classification system are not exclusive for perinatal medicin, but can easily be used in other fields of medicin.

Although not in concordance with the concept of this classification system of SSF’s, it was agreed upon by the CDG to classify the patient factor in category 8 “other”. It can be argued, that the role of the patient in the occurrence of a substandard factor will become evident when a substandard factor is analysed and therefore the patient factor should not be incorporated in the classification. However, the methodical analyses of SSF’s is a time consuming process and in-depth analysis is therefore not always feasible. Both the CDG and the ICG felt that it would be a loss of important information if the patient factor was not somehow incorporated in the classification. Especially, because the patient factor may be the result of insufficient information given to the patient by the caregiver.

The chance corrected inter-rater agreement, as indicated by the κ values, between and within groups was good, moderate and poor for the main category, subcategory one and subcategory two respectively with the ICG scoring lower than the CDG. Because of the confidentiality of the audit meetings case notes were not available. We found that the main problem for the classification groups was that the SSF were often inadequately phrased given the situation of the unknown context. This may be improved by training the participants of the audit meetings in phrasing the SSF correctly. The structure of the classification may even help the caregivers to phrase the substandard factor in an understandable way. For example: in our database the SSF that is phrased as “ Blood pressure with epidural” may be rephrased into “the guideline for taking the blood pressure in a patient with epidural analgesia is not followed” This phrasing makes it clear, that the SSF concerns the use of the guidelines for a diagnostic medical procedure. This makes it easy to classify to the category “medical practice, diagnosis (hypo or hypertension), use of guidelines (7.1.1)

Despite the problems with the phrasing of substandard factors we demonstrated however that, even with crude methods, such as simple counts irrespective of agreement and with uncorrected agreement scores, patterns in the occurrence of SSF can be recognised with our classification system.

As in all classification systems unclassifiable SSF may be considered a loss of information and a loss of opportunity to improve care. In our classification system the percentage of unclassifiable SSF was only 3 %. We expect that this percentage will be reduced with the improvement of the phrasing of the SSF’s.

## Conclusions

284 SSF identified in the northern region of the Netherlands during perinatal audit meetings allowed a multidisciplinary team to develop a classification system for substandard factors in perinatal care. The categories in the classification are well defined, the guidelines and the examples are clear. The multidisciplinary inter-rater agreement is moderate to good. With a short training and the use of the guidelines the classification can easily be used by a multidisciplinary group of caregivers. Improvement of the phrasing of the SSF is expected to improve the inter-rater agreement. Further testing of the inter-rater agreement outside our region will give opportunity to enhance and refine our classification system.

## Additional file

Additional file 1:
**Examples of the classification of substandard factors.** (PDF 37 kb)

## Abbreviations

CDG: Classification development group
ICG: Independent classification group
SSF: Substandard factor
