# Supplementary material for: Classification of substandard factors in perinatal care: development and multidisciplinary inter-rater agreement of the Groningen-system
Source: BMC Pregnancy Childbirth. 2015 Sep 11;15:215. doi: 10.1186/s12884-015-0638-5 (PMC4567791; doi:10.1186/s12884-015-0638-5)
Supplement: Additional file 1: — Examples of the classification of substandard factors. (PDF 37 kb) [file 12884_2015_638_MOESM1_ESM.pdf]

Appendix A Examples of the classification of substandard factors

1 Equipment and Materials

Example1: **use**

SSF: In a very obese patient the blood pressure was taken with a narrow cuff instead of a broad cuff, which was also available.
Reasoning: Inappropriate equipment was used for taking the blood pressure, so the equipment was not used as intended.
Classification: 1.1

Example 2: **performance**
SSF: Defect in the Ambu bag in the resuscitation set.
Reasoning: The equipment is available, but is faulty and does not function properly.
Classification: 1.2

Example 3: **availability**

SSF: There was no equipment for neonatal resuscitation on the adult ICU when a premature baby was born to a mother on ventilation.
Reasoning: The equipment for resuscitation are available, but not at the location where they were needed.
Classification: 1.3

Example 4: **other**
SSF: The CTG monitor does not work.
Reasoning: It is unclear if the monitor itself 1.2 is not functioning or for example the scalp electrode 1.3.
Classification: 1.4

2 Medication

Example 1: **substance itself**
SSF: Instead of a uterine relaxant drug a uterine stimulating drug was given.
Reasoning: Incorrect medication was given.
Classification: 2.1

Example 2: **dosage**
SSF: Incorrect dosage of pain relief medication was given.
Reasoning: The recommended dosage was not administered
Classification: 2.2

Example 3: **administration**
SSF: A uterine stimulating drug was given intramuscularly instead of intravenously.
Reasoning: the recommended route of administration was not used in the given clinical situation.
Classification: 2.3

Example 4: **other**
SSF: Medication
Reasoning: It is clear that something went wrong with the medication, but it is unclear what went wrong.
Classification: 2.4

3 Additional tests/ investigations

Example 1: request form
SSF: Insufficient clinical information on pathology(PA) request form
Reasoning: The request form was not filled out correctly
Classification: 3.2

Example 2: **labelling patient material**

SSF: CTG in wrong patient's file
Reasoning: This can be compared with a wrong patient label on a blood test tube.
Classification: 3.2

Example 3: **transport patient material**
SSF: The placenta was sent to the pathologist in the wrong transport medium
Reasoning: Patient material was transported to the laboratory incorrectly.
Classification: 3.3

Example 4: **execution**
SSF: Amniotic fluid for genetic tests was collected vaginally and not by amniocentesis
Reasoning: Genetic tests and tests in general start with the correct collection of material to perform the tests on. Amniotic fluid collected vaginally is contaminated with maternal cells and cannot be used for genetic tests.
Classification: 3.4

Example 5: **interpretation test result by test performer**
SSF: CTG misinterpreted as normal

Reasoning:The CTG is a diagnostic test for the assessment of fetal wellbeing. The interpretation of the test by the caregiver is part of the test.
Classification: 3.5

Example 6: **test result to person who requested**
SSF: The test result was not communicated to the person who requested the test within the standard 3 days, but after 7 days.
Reasoning: There was a delay in communicating the test result to the person who requested the test
Classification: 3.6

Example 7: **other**
SSF: Test result not communicated
Reasoning: It is unclear to whom the test result should have been communicated, but we know that the SSF is about testresults.
Classification: 3.7
Note: Example 6 en 7 could also be classified as 6.5., but category 3 (additional tests/investigations) precedes category 6 (communication) in the hierarchy and should be used.

4 Transportation

Example 1: **home-hospital**
SSF: Delay arrival at the hospital after referral to gynaecologist.
Reasoning: The recommendations for transport of a patient to the hospital in time are not followed (it took more time than expected).
Classification: 4.1

Example 2: **between hospitals**
SSF: The ambulance with the neonatologist took more than 2 hours to get from the general hospital to the tertiary centre (20 minutes ride).
Reasoning: The recommendations for transport of a patient between hospitals in time are not followed.
Classification: 4.2

Example 3: **within the hospital**
SSF: Intravenous drip and nasal tube displaced when baby was changed from transport incubator to normal incubator.
Reasoning: Changing patients from one bed to another can be considered as transportation within the hospital
Classification:4.3

Example 4: **other**
SSF: Transportation of the patient is performed according to the guidelines.
Reasoning: It is clear that the SSF involved transportation, but unclear what kind of transportation and from where to where.
Classification: 4.4

5 Documentation

Example 1: **basic data**
SSF: incomplete documentation of the history (2^{e} child has metabolic disorder, although caregiver had knowledge of this family history it was not documented in patient record.
Reasoning: Information that is gathered by history taking can be considered as routinely collected data and are therefore basic data.
Classification:5.1

Example 2: **observations/examinations**
SSF: There is no record of the neonatal examination, but it is clear that the examination was performed.
Reasoning: The neonatal examination was not documented.
Classification:5.2

Example 3: **considerations/management**
SSF: The indication to perform a CS is not documented in the patient record.
Reasoning: The argumentation for this management is unclear and not recorded in the patient record
Classification: 5.3

Example 4: **other**
SSF: Documentation
Reasoning: It is clear that this SSF involved documentation, but unclear what kind of documentation
Classification: 5.4

6 Communication

Example 1: **same echelon, equal level**
SSF: Referral from general practitioner to the community midwife without patient record.
Reasoning: Although of different disciplines, the midwife and the general practitioner are of an equal level with respect to maternity care.
Classification: 6.1

Example 2: **same echelon, different level**
SSF: No consultation of the gynaecologist by ob-gyn nurse about telephone consultation of patient with decreased fetal movements and hypertension.
Reasoning: In this situation the ob-gyn nurse and the gynaecologist are of unequal level

Classification: 6.3

Example 3**: different echelons**
SSF:The gynaecologist did not report back to the community midwife after referral of her patient.
Reasoning: The caregiver in the first echelon was not informed by the caregiver in the 2nd echelon
Classification: 6.4

Example 4: **with the patient**
SSF: Telephone consultation was not done although promised to the patient
Reasoning: This is about communication between patient and caregiver
Classification: 6.5

Example 5: **between departments**
SSF: Unclear agreements on breastfeeding between the obstetric and the neonatal departments
Reasoning: The communication between departments is insufficient
Classification: 6.6

Example 6: **other**
SSF: Letters are not always sent to all involved caregivers
Reasoning: It is clear that this is about written communication, but it is not clear between which caregivers
Classification: 6.7

Note: If letters are not written the classification would be documentation.

7 Medical practice

Example 1a: **diagnosis, use of guidelines**
SSF: The glucose tolerance test(GTT) was performed too late.
Reasoning: The GTT can be considered to be a diagnostic test which has to be performed at a certain time in pregnancy. This is laid down in a guideline.
Classification: 7.1.1

Example 1b: **diagnosis, content of guidelines**
SSF's: Paediatricians and community midwives have different guidelines for the diagnosis of neonatal hyperbilirubinaemia.
Reasoning: There is a discrepancy between guidelines for the same problem. This is about the content of guidelines.
Classification: 7.1.2

Example 1c: **diagnosis, common practice**
SSF: Incomplete history taken at booking
Reasoning: The medical and social history can be considered to be a diagnostic tool. The result determines the management of the pregnancy. It is common practice to take a complete history.
Classification: 7.1.3

Example 2a: **management plan and management, use of guidelines**
SSF: The patient is already 15 weeks pregnant when she comes for booking of the pregnancy.
Reasoning: This means that the patient did not get the care she should have had. The guideline for a management plan for the early pregnancy could not be followed. (The reason why she came too late is not relevant for the classification)
Classification: 7.2.1

Example 2c: **management plan and management, content of guidelines**
SSF: Contradicting advice in the guidelines for breastfeeding in the maternity and neonatology department.
Reasoning: staff taking care of and advising the same mother use contradicting guidelines
Classification: 7.2.2

Example 2c: **management plan and management, common practice**
SSF: patient with complex pathology not presented at the multidisciplinary meeting
Reasoning: It is part of the management plan and common practice to present patients with complex pathology at this meeting to evaluate the management plan and make decisions for future management.
Classification: 7.2.3

8 Other

There is sufficient information to allocate the substandard factor, but the substandard factor cannot be allocated to the preceding categories.
Remark: Usually it is the patient who behaved unexpectedly or changed the management plan herself. Even in these cases insufficient information by professionals needs to be considered. However for the time being patient factors are classified 8.

9 Non classifiable

There is insufficient information to allocate the substandard factor.
